# Survival after blinatumomab treatment in pediatric patients with relapsed/refractory B-cell precursor acute lymphoblastic leukemia

**DOI:** 10.1038/s41408-018-0117-0

**Published:** 2018-08-22

**Authors:** Lia Gore, Franco Locatelli, Gerhard Zugmaier, Rupert Handgretinger, Maureen M. O’Brien, Peter Bader, Deepa Bhojwani, Paul-Gerhardt Schlegel, Catherine A. Tuglus, Arend von Stackelberg

**Affiliations:** 1 University of Colorado School of Medicine and Children’s Hospital Colorado, Aurora, CO USA; 2 Ospedale Pediatrico Bambino Gesù, Rome, Italy; 3 University of Pavia, Pavia, Italy; 4 Amgen Research (Munich), Munich, Germany; 5 University of Tübingen, Tübingen, Germany; 6 Cincinnati Children’s Hospital Medical Center, University of Cincinnati School of Medicine, Cincinnati, OH USA; 7 Division for Stem Cell Transplantation and Immunology, Department of Children and Adolescents, University Hospital Frankfurt, Goethe University, Frankfurt, Germany; 8 Children’s Hospital of Los Angeles, Los Angeles, CA USA; 9 University Children’s Hospital Würzburg, Würzburg, Germany; 10 Amgen Inc., Thousand Oaks, CA USA; 11 Charité Campus Virchow, Berlin, Germany

Acute lymphoblastic leukemia (ALL) is the most common cancer in children^1^. Pediatric patients with disease refractory to last chemotherapy, relapse after allogeneic hematopoietic stem-cell transplantation (alloHSCT), or second or further relapse have a particularly poor prognosis^2^. Intensive chemotherapy followed by alloHSCT after achieving remission can result in cure for some patients. However, survival is still low with this approach^3^. Thus, additional treatment modalities with acceptable toxicity are needed to improve long-term survival.

Blinatumomab is a bispecific T-cell engager (BiTE) antibody construct that directs CD3-positive effector memory T-cells to CD19-positive target leukemia cells. We previously reported the primary results of a phase 1–2 study of blinatumomab treatment in pediatric patients with relapsed/refractory B-cell precursor ALL (BCP-ALL)^4^. In that report, of the 70 patients who received the recommended phase 2 dose in either phase, 27 patients (39%; 95% confidence interval [CI], 27 to 51%) achieved complete remission (CR) within the first two cycles; of these, 52% attained negative minimal residual disease (MRD). Adverse events of interest included neurologic events (24%), cytokine release syndrome (11%), and transient, clinically non-significant elevation of alanine aminotransferase (19%), aspartate aminotransferase (14%), or bilirubin (6%). The objective of this follow-up analysis is to examine the final results for remission and survival 24 months after blinatumomab treatment.

Full study methods have been reported previously^4^. In brief, this open-label, single-arm phase 1–2 study enrolled patients ≤ 18 years of age with relapsed/refractory BCP-ALL and > 25% bone marrow blasts if disease was primary refractory or in refractory first relapse after full standard induction regimen, in second or further relapse, or in any relapse after alloHSCT. Patients in nonrefractory first relapse without prior alloHSCT were not permitted in the study. All patients received blinatumomab as a 4-week continuous intravenous infusion, with a 2-week treatment-free interval after each cycle. On the basis of phase 1 results, the recommended phase 2 dose was 5 µg/m^2^/day for the first week of cycle 1, followed by 15 µg/m^2^/day for the remaining 3 weeks of cycle 1 and for all 4 weeks of the following cycles. Infusions were administered in the hospital during the first week of cycle 1 and the first 2 days of cycle 2, then in an outpatient setting whenever clinically appropriate for a given patient’s condition. Bone marrow aspirate for response assessment (or biopsy if aspirate could not be obtained) was performed during screening, on day 15 of cycle 1, and on day 29 of each cycle. Patients achieving hematologic CR within the first 2 cycles could receive up to three additional cycles and/or consolidation chemotherapy and/or alloHSCT at any time per investigator’s choice.

Central nervous system prophylaxis according to institutional/national standards was administered at age-adjusted doses when bone marrow assessment was performed. Patients who experienced neurologic adverse events requiring medical intervention were given dexamethasone at 0.2 to 0.4 mg/kg/day (maximum, 24 mg/day) for up to 3 days and anti-seizure medications as indicated.

Twenty-six European and US centers conducted this study. Institutional review boards and independent ethics committees approved the study protocol. Patients’ legal representatives gave written informed consent and patients provided assent as appropriate, according to institutional guidelines. The study was registered at www.clinicaltrials.gov (NCT01471782) and conducted according to the Principles of the Declaration of Helsinki and rules of Good Clinical Practice.

Patients enrolled in this study had baseline characteristics that implied a dismal prognosis^4^. Relapse within 6 months after last prior treatment attempt before start of blinatumomab treatment occurred in 50 of 70 patients (71.4%) who received the recommended phase 2 dose. This reflected the typically short duration of prior remission, which is a major adverse prognostic factor^5^. Thirty-nine (55.7%) patients had refractory disease, 40 (57.1%) had undergone prior alloHSCT before blinatumomab treatment, 52 (74.3%) had a blast count in bone marrow of 50% or more, and 10 (14.3%) had MLL translocation.

Overall survival by alloHSCT use before blinatumomab was estimated using the Kaplan–Meier method. Overall survival by alloHSCT use after blinatumomab was estimated using Simon-Makuch analyses^6^ with a 45-day landmark (i.e., after cycle 1). In addition, overall survival by alloHSCT use after blinatumomab was assessed in the 27 patients with CR within the first two cycles, using the Mantel-Byar method. Overall survival by complete MRD response was estimated using the Kaplan–Meier method with a 15-day landmark (i.e., the first MRD assessment post-baseline).

This report includes final results for the 70 patients who received the recommended dose of blinatumomab 5/15 µg/m^2^/day (Table 1). Fourteen (20.0%) patients were alive at the final 24-month follow-up visit. Eight (11.4%) additional patients were alive at the time of study discontinuation because of withdrawal or loss to follow-up. Thus, 22 of 70 (31.4%) patients treated at the recommended phase 2 dose were alive at their last follow-up visit. Of the 48 (68.6%) patients who died on study, 3 died in continuous CR after blinatumomab, all due to transplant-related complications, 15 achieved CR with blinatumomab and subsequently relapsed, and 30 did not achieve CR with blinatumomab.

**Table 1:** Outcomes among pediatric patients treated with blinatumomab 5/15 µg/m^2^/day

Outcome subject	Age [years]	Disease status^a^: prior alloHSCT/# relapses/refractory	Outcomes and other treatments after blinatumomab					
			CR/MRD response	CD19-negative	AlloHSCT	CAR T-cells	Disposition	Survival [months]
Alive at last follow-up (survivors at 24 months)								
1	1	Yes/-/No	Yes/Yes		Yes		Alive	24.0
2	1	Yes/-/No	Yes/Yes		Yes		Alive	24.3
3	14	Yes/-/Yes	Yes/Yes				Alive	23.6
4	13	Yes/-/No	Yes/No				Alive	24.4
5	17	No/2 + /No	Yes/No		Yes		Alive	24.1
6	1	Yes/-/No	Yes/No			Yes	Alive	22.8
7	11	Yes/-/No	No/No		Yes		Alive	24.4
8	9	Yes/-/No	No/No		Yes		Alive	24.1
9	17	Yes/-/No	No/No		Yes		Alive	23.7
10	12	No/1/Yes	No/No		Yes		Alive	23.5
11	5	Yes/-/Yes	No/No				Alive	24.3
12	17	Yes/-/No	No/No		Yes	Yes	Alive	23.8
13	< 1	No/0/Yes	No/No	Yes	Yes		Alive	23.7
14	17	No/2 + /No	No/No		Yes	Yes	Alive	23.8
Discontinued study early, alive at time of discontinuation								
15	6	No/2 + /No	Yes/Yes	Yes	Yes		Alive: Subject withdrew	10.9^b^
16	14	Yes/-/No	Yes/Yes				Alive: Subject withdrew	9.9^b^
17	9	Yes/-/Yes	Yes/No				Alive: Subject withdrew	1.8^b^
18	1	No/1/Yes	No/Yes				Alive: Subject withdrew	0.5^b^
19	5	No/1/Yes	No/NA				Alive: Subject withdrew	0.4^b^
20	5	Yes/-/No	No/No		Yes		Alive: Lost to follow-up	12.0^b^
21	7	Yes/-/No	No/No				Alive: Physician decision	0.5^b^
22	9	No/1/Yes	No/No				Alive: Subject withdrew	0.9^b^
Died while in continuous hematological CR after blinatumomab								
23	1	No/2 + /Yes	Yes/Yes		Yes		Died: multiorgan failure	3.1
24	10	Yes/-/Yes	Yes/Yes	NA	Yes		Died: fatal septic shock	12.5
25	16	Yes/-/Yes	Yes/Yes		Yes		Died: renal failure	14.6
Achieved hematological CR and relapsed before death								
26	2	Yes/-/No	Yes/Yes	NA			Died	4.9
27	5	No/1/Yes	Yes/Yes	Yes^c^			Died	10.4
28	8	Yes/-/No	Yes/Yes		Yes		Died	19.4
29	8	Yes/-/No	Yes/Yes	NA			Died	15.0
30	7	Yes/-/Yes	Yes/Yes		Yes		Died	17.3
31	12	Yes/-/Yes	Yes/Yes		Yes		Died	3.2
32	6	No/1/Yes	Yes/Yes		Yes		Died	6.5
33	3	No/2 + /Yes	Yes/NA		Yes	Yes	Died	8.1
34	11	Yes/-/No	Yes/No				Died	6.5
35	10	Yes/-/No	Yes/No				Died	9.3
36	1	Yes/-/No	Yes/No				Died	1.7
37	16	Yes/-/No	Yes/No			Yes^d^	Died	3.6
38	1	Yes/-/No	Yes/No				Died	3.7
39	6	No/1/Yes	Yes/No				Died	5.2
40	4	No/1/Yes	Yes/No		Yes	Yes	Died	11.2
Never responded to blinatumomab before death								
41	7	Yes/-/No	No/NA				Died	0.2
42	11	No/1/Yes	No/NA				Died	0.8
43	13	No/1/Yes	No/NA				Died	0.5
44	6	No/1/Yes	No/NA		Yes		Died	2.7
45	14	Yes/-/No	No/NA				Died	0.7
46	3	Yes/-/Yes	No/NA				Died	0.8
47	12	Yes/-/No	No/No				Died	1.7
48	5	No/0/Yes	No/No				Died	2.9
49	3	No/1/Yes	No/No				Died	4.3
50	9	No/1/Yes	No/No				Died	4.0
51	13	No/2 + /Yes	No/No		Yes		Died	12.4
52	14	No/1/Yes	No/No				Died	2.8
53	< 1	No/1/Yes	No/No	Yes			Died	1.1
54	17	Yes/-/No	No/No		Yes		Died	8.2
55	4	Yes/-/Yes	No/No				Died	4.2
56	5	Yes/-/No	No/No				Died	11.8
57	12	Yes/-/Yes	No/No				Died	1.4
58	8	Yes/-/Yes	No/No				Died	2.4
59	16	Yes/-/No	No/No				Died	1.4
60	9	No/1/Yes	No/No				Died	1.6
61	11	Yes/-/No	No/No				Died	10.6
62	6	No/1/Yes	No/No		Yes		Died	15.8
63	10	No/2 + /Yes	No/No				Died	3.8
64	6	No/2 + /No	No/No				Died	3.7
65	6	No/1/Yes	No/No				Died	3.5
66	< 1	No/1/Yes	No/No				Died	2.3
67	13	Yes/-/Yes	No/No			Yes	Died	7.5
68	11	No/1/Yes	No/No				Died	5.7
69	10	Yes/-/Yes	No/No				Died	1.6
70	8	Yes/-/No	No/No				Died	9.6

*alloHSCT* allogeneic hematopoietic stem cell transplantation, *CR* complete remission, *MRD* minimal residual disease, *NA* not analyzed, *OS* overall survival

^a^Based on reason for study inclusion; number of prior relapses was not reported for patients with prior alloHSCT, who could enroll regardless of prior relapses or refractory disease

^b^Overall survival was censored at the time of early study discontinuation

^c^For this patient, CD19-negativity occurred for the second relapse after blinatumomab treatment

^d^This patient received CAR T-cells before blinatumomab treatment; all other reported CAR T-cell administrations occurred after blinatumomab treatment

The 14 survivors at the 24-month follow-up visit by baseline age included 4 of 10 (40.0%) patients aged < 2 years, 1 of 20 (5.0%) patients aged 2 to 6 years, and 9 of 40 (22.5%) patients aged 7 to 18 years. Six of 10 patients aged < 2 years in this analysis had an MLL translocation; 3 of 6 completed the 24-month follow-up visit. These three survivors aged < 2 years with an MLL translocation included one who achieved hematologic CR and negative MRD before receiving alloHSCT in continuous CR, one who did not achieve hematologic CR and received subsequent alloHSCT, and one who achieved hematologic CR but continued to have MRD and did not receive subsequent alloHSCT.

**Fig. 1:**
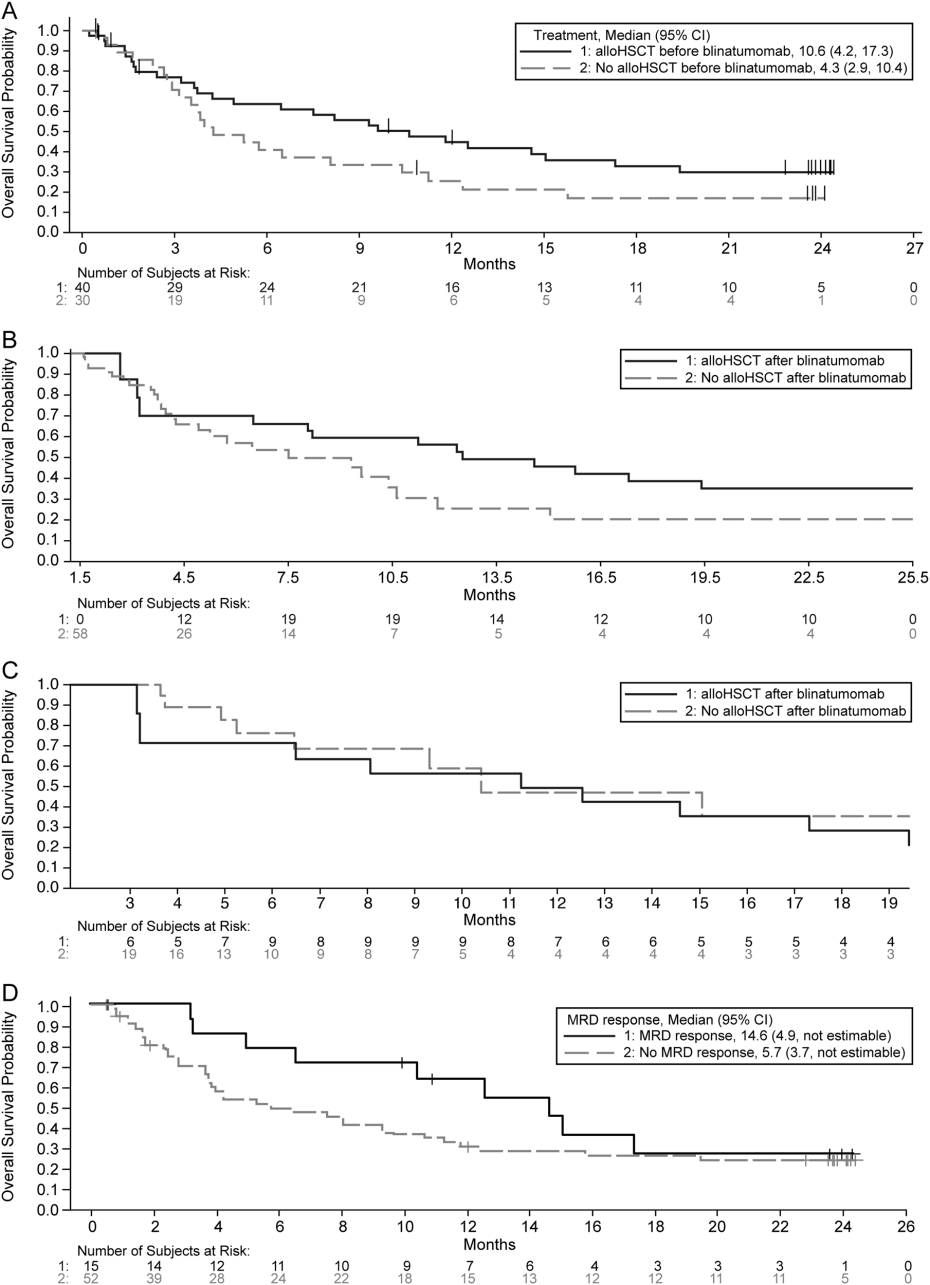
**Overall survival after blinatumomab treatment in pediatric patients with relapsed/refractory B-cell precursor acute lymphoblastic leukemia.** **a** Kaplan–Meier analysis of overall survival after initiation of blinatumomab treatment, according to the use of alloHSCT before blinatumomab, among all patients who received the recommended dosage. **b** Simon-Makuch analyses of overall survival after initiation of blinatumomab treatment, using a 1.5-month (45-day) landmark, according to use of alloHSCT after blinatumomab. **c** Simon-Makuch analyses of overall survival after initiation of blinatumomab treatment, using a 3-month (85-day) landmark, according to use of alloHSCT after blinatumomab in the subset of patients with CR within the first two cycles. **d** Overall survival by achievement of MRD response after blinatumomab treatment. MRD was not assessed for one patient in hematological CR. alloHSCT allogeneic hematopoietic stem-cell transplantation, CR complete remission, MRD minimal residual disease

The 24-month Kaplan–Meier estimate for overall survival was 25% and median overall survival was 7.5 months (95% CI, 4.0 to 11.8). Although patients with relapse after alloHSCT generally have a poor outcome, prior alloHSCT was associated with prolonged survival after blinatumomab treatment (Fig. 1a): median overall survival was 10.6 months (95% CI, 4.2 to 17.3) for patients who received prior alloHSCT compared with 4.3 months (95% CI, 2.9 to 10.4) for those who did not (*p* = 0.1414).

Twenty-five (35.7%) patients received alloHSCT after blinatumomab treatment, including 13 after blinatumomab-induced CR and 12 nonresponders to blinatumomab. Before blinatumomab treatment, 6 of 12 nonresponders had relapsed after alloHSCT and the other 6 were refractory to treatment; all 12 were responsive to chemotherapy and subsequent alloHSCT after blinatumomab. The median time to alloHSCT was 1.84 months. In a Simon-Makuch analysis, overall survival appeared to be longer in patients who received alloHSCT compared with those who did not (Fig. 1b). In a Mantel-Byar analysis of overall survival among the 27 patients who achieved CR within the first two cycles, the odds ratio for death for alloHSCT after blinatumomab vs. no alloHSCT after blinatumomab was 1.26 (95% CI, 0.47 to 3.42; Fig. 1c).

The population of transplanted responders was heterogeneous. Eight patients underwent transplantation in continuous CR without additional antileukemic treatment, three received additional chemotherapy in continuous CR after blinatumomab treatment before alloHSCT, and two underwent alloHSCT after relapse. The patients who received alloHSCT while in continuous CR without additional therapy between blinatumomab and alloHSCT were in late stage of disease. Six of the 8 patients had already undergone alloHSCT at least once before starting blinatumomab treatment. In addition, 6 of the 8 patients were refractory to chemotherapy before blinatumomab treatment; for the other two patients, alloHSCT after blinatumomab was their third alloHSCT. Among the eight patients, blinatumomab was administered as 3rd-line treatment for four patients, 4th-line treatment for three patients, and 5th-line treatment for one patient. One of 8 patients completed the 24-month follow-up visit. It is challenging to find published literature for the role of alloHSCT in a mostly refractory population. With multiagent chemotherapy, 2-year survival from achievement of CR after 4th-line treatment is 13%^2^, which is comparable to our data, but this does not reflect the high number of subjects with refractory disease in our population.

Complete MRD response was associated with a median overall survival of 14.6 months and absence of MRD response was associated with a median overall survival of 5.7 months (Fig. 1d). Sixty-seven patients treated at the phase 2 dose had CD19 assessment after blinatumomab treatment. Four (6.0%) of the 67 patients were CD19-negative after blinatumomab treatment: two responders had a CD19-negative relapse, which was described elsewhere in detail;^7^ and two nonresponders did not express CD19 after blinatumomab treatment.

Six patients received chimeric antigen receptor (CAR) T-cells after blinatumomab and one received CAR T-cells before blinatumomab. All seven patients were CD19-positive after blinatumomab treatment. Of the six patients who received CAR T-cells after blinatumomab, three were alive in CR at last follow-up, two initially responded to blinatumomab and died after relapse, and one received CAR T-cells as a non-responder to blinatumomab and died after CAR T-cell treatment. The patient who had received CAR T-cells prior to blinatumomab treatment was refractory to blinatumomab and died. A multicenter, global, pivotal, registration study investigated the CAR T-cell therapy, tisagenlecleucel, in children and young adults with relapsed/refractory BCP-ALL^8^. Of the 75 patients who received CAR T-cells in that study, 81% (95% CI, 71 to 89%) achieved CR or CR with incomplete hematologic recovery within 3 months, all of whom were negative for MRD. Cytokine release syndrome was grade 3 in 21% of patients and grade 4 in 25% of patients. Neurologic events were grade 3 in 13% of patients. Grade 3 or 4 adverse events were suspected to be related to CAR T-cells in 73% of patients. In the CAR T-cell study, the minimum blast count in bone marrow at enrollment was 5%; in our blinatumomab study, it was 25%.

Detailed safety results for our blinatumomab study were reported previously^4^. Three (4.3%) patients experienced grade 3 neurologic events; no grade 4 or 5 neurologic events occurred. Two (2.9%) patients interrupted blinatumomab treatment because of grade 2 neurotoxicity (i.e. seizures) and restarted blinatumomab after the event resolved: 1 achieved CR with incomplete recovery of peripheral blood counts but died of disease progression at 8.1 months and the other was a non-responder (did not qualify for CR, partial remission, or progressive disease) but was alive when lost to follow-up at 12 months. Grade 3 cytokine release syndrome occurred in 3 (4.3%) patients and grade 4 cytokine release syndrome in 1 (1.4%) patient: all four patients achieved CR with blinatumomab (one with full recovery of peripheral blood counts, one with incomplete recovery of peripheral blood counts, and two with neither complete nor incomplete recovery of peripheral blood counts). None of the four patients were alive at the end of the 2-year follow up: one died in remission (renal failure) and three relapsed before death.

In conclusion, in this final analysis of the first study of blinatumomab in pediatric patients with relapsed/refractory BCP-ALL, alloHSCT before or after blinatumomab was associated with a positive effect on overall survival. There was no difference in overall survival between transplanted and non-transplanted responders. No firm conclusion can be drawn because of the low sample size of 13 transplanted responders, including five who received alloHSCT after additional antileukemic treatment or relapse. There was also a trend of prolonged survival among patients who achieved complete MRD response. Six patients received CAR T-cells after blinatumomab; 3 of 6 were alive at the last assessment. Larger data sets with longer follow-up are warranted in late or final stages of disease to evaluate the role of complete MRD response, the replacement of transplantation by treatment with blinatumomab or alternative treatment, and the feasibility of CAR T-cell administration after or before blinatumomab treatment.
